# Subjective perception of treatment in patients first referred to radiotherapy and its relationship to their well-being

**DOI:** 10.1192/j.eurpsy.2021.1159

**Published:** 2021-08-13

**Authors:** M. Kovyazina, E. Rasskazova, A. Kuznetsova, R. Shilko, Y. Zinchenko, V. Sadovnichaja

**Affiliations:** 1 Clinical Psychology, Moscow State University, Moscow, Russian Federation; 2 Faculty Of Psychology, Lomonosov Moscow state University, Moscow, Russian Federation; 3 Faculty Of Psychology, Lomonosov Moscow State University, Moscow, Russian Federation

**Keywords:** radiotherapy, treatment representation, well-being

## Abstract

**Introduction:**

There are wide-spread fears and expectations about radiotherapy in people referred to it that are not only unrealistic (Shaverdian et al., 2018) but also lead to poorer compliance with doctors and poorer satisfaction with treatment (Dong et al., 2014).

**Objectives:**

The aim was to reveal relationship between different aspects of subjective perception of radiotherapy in patients and their well-being.

**Methods:**

34 patients first referred to radiotherapy, 23-70 years old (mostly females with breast cancer) filled modified version of Beliefs about Medication Questionnaire including items about radiotherapy (Horne et al., 1996), Satisfaction With Life Scale (Diener et al., 1985), Scale of Positive And Negative Experience (Diener et al., 2009).

**Results:**

Six scales were revealed by factor analysis in the structure of beliefs about radiotherapy (Cronbach’s alphas .74-.85): confidence in the effectiveness of radiation therapy, subjective need for it, lack of understanding of it, concern and general negative attitudes towards radiotherapy, doubts about the effectiveness of radiation therapy. Elder patients reported higher need for radiotherapy but also higher concerns about it (r=.35-.37). Concerns about radiotherapy were related to lower satisfaction with life and positive emotions (r=-.44 - -.34) while subjective need of radiotherapy was related to higher health anxiety (r=.71) and lower positive emotions (r=-.41).

**Conclusions:**

Subjective concerns of patients regarding radiotherapy are related to poorer well-being and could be addressed in psychotherapy.

